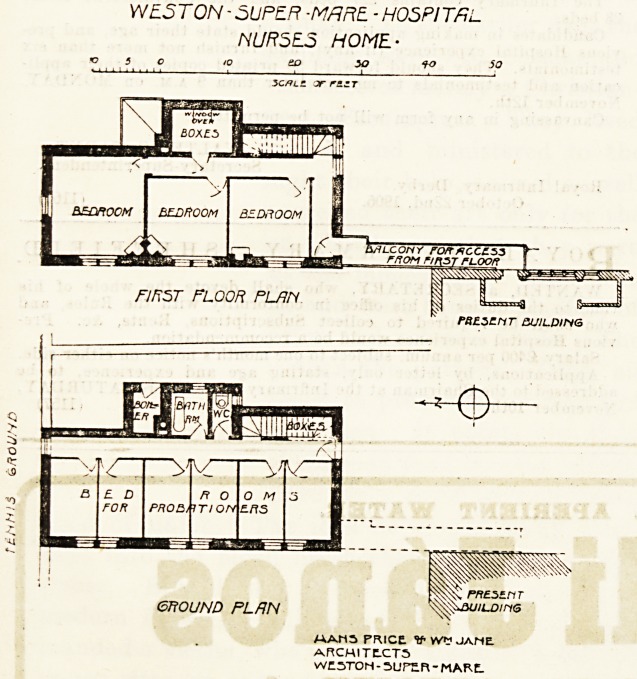# Nurses' Home at the Weston-Super-Mare Hospital

**Published:** 1906-10-27

**Authors:** 


### NURSES' HOME AT THE WESTON-SUPER-MARE HOSPITAL.

Most of the older hospitals have realised that the accommodation provided for their nursing staffs was insufficient and new "Homes" have lately been added to many institutions. Weston-super-Mare has followed suit, and in December last a new block containing the much-needed provision was formally opened by the President of the Board of Management, Mr. E. E. Baker.

The new wing is connected by a covered bridge to the north-east corner of the old building. On the ground floor it contains six bedrooms for probationers. These rooms are approached from a corridor which runs right through the block, and the corridor is lighted and ventilated by a window at each end. On the east side of the corridor are the staircase, bath-room, closet, and boiler-room. The first floor has three good bedrooms for nurses and a box-room.

The addition cannot fail to be a great source of comfort to the members of the nursing staff, and although the block is a small one it contains everything that is necessary for the numbers, and all available space has been cleverly appropriated by the architects employed, who were Messrs. Price and Jane. The contractors were Messrs. G. and E. Stokes. The cost of the annexe was £850, exclusive of furniture, for which £150 was allowed, so that with the covered bridge, adjuncts, and furniture the cost per room did not much exceed £100.

## Figures and Tables

**Figure f1:**